# PD-L1 expression complements CALGB prognostic scoring system in malignant pleural mesothelioma

**DOI:** 10.3389/fonc.2023.1269029

**Published:** 2023-12-04

**Authors:** Alejandro Avilés-Salas, Luis Cabrera-Miranda, Norma Hernández-Pedro, Diana Sofía Vargas-Lías, Suraj Samtani, Wendy Muñoz-Montaño, Daniel Motola-Kuba, Luis Corrales-Rodríguez, Claudio Martín, Andrés F. Cardona, Cittim B. Palomares-Palomares, Oscar Arrieta

**Affiliations:** ^1^ Pathology Department, Instituto Nacional de Cancerología (INCan), Mexico City, Mexico; ^2^ Thoracic Oncology Unit, Instituto Nacional de Cancerología (INCan), Mexico City, Mexico; ^3^ Personalized Medicine Laboratory, Instituto Nacional de Cancerología (INCan), Mexico City, Mexico; ^4^ Medical Oncology Department, Clínica Las Condes Santiago, Santiago, Chile; ^5^ Departments of Biomedical Research and Gastroenterology and Liver Unit and Hemodialysis Unit, Medica Sur Clinic and Foundation, Mexico City, Mexico; ^6^ Medical Oncology Department, San Juan de Dios Hospital, San José, Costa Rica; ^7^ Department of Medicine, Western University, London, ON, Canada; ^8^ Thoracic Oncology Unit and Direction of Research, Science and Education, Luis Carlos Sarmiento Angulo Cancer Treatment and Research Center (CTIC), Bogotá, Colombia; ^9^ Clinical and Translational Oncology Group, Clínica del Country, Bogotá, Colombia; ^10^ Molecular Oncology and Biology Systems Research Group (Fox-G), Universidad El Bosque, Bogotá, Colombia

**Keywords:** PD-L1, CALGB, mesothelioma, prognostic factor, immunohistochemistry

## Abstract

**Background:**

Programmed death ligand-1 (PD-L1) expression is a predictive biomarker in patients with lung cancer, but its role in malignant pleural mesothelioma (MPM) remains unclear. Evidence suggests that higher PD-L1 expression is correlated with worse survival. CALGB is the main scoring system used to predict the benefit of chemotherapy treatment. This study aimed to determine the prognostic value of PD-L1 expression and its addition to CALGB scoring system in patients with MPM.

**Methods:**

In this retrospective analysis, we evaluated samples with confirmed locally advanced or metastatic MPM. PD-L1 Tumor Proportional Score (TPS) was determined by immunohistochemistry at diagnosis.

**Results:**

73 patients were included in this study. A cutoff value of 15 was set for a high or low PD-L1 TPS. In total, 71.2% (n=52) and 28.8% (n=21) of individuals harbored low or high PD-L1 expression, respectively. PD-L1^High^ was associated with worse median progression-free Survival (mPFS) [4.9 vs. 10.8 months; HR 2.724, 95% CI (1.44-5.14); p = 0.002] and Overall Survival (OS) [6.0 vs. 20.9 months; HR 6.87, 95% CI (3.4-8.7); p<0.001] compared to patients with PD-L1^Low^. Multivariate analysis confirmed that PD-L1 expression was an independent factor for PFS and OS in patients with MPM and CALGB score of 5-6.

**Conclusion:**

PD-L1 addition to CALGB scale improves its prognostic estimation of MPM survival and should be considered in future research.

## 1 Introduction

Malignant pleural mesothelioma (MPM) is an underreported neoplasm with unknown incidence in Mexico (1). This disease founds its onset on pleura cells, and it is characterized by epithelioid (85.4%), sarcomatoid (12.2%), or biphasic (2.4%) histologies (1). Asbestos (2) and erionite (3) exposure are the main risk factors for MPM development, with a latency of 40-50 years between exposure and disease. Most MPM cases are diagnosed at advanced stages, which results in unfavorable responses to surgery and platinum-based chemotherapy, and short survival outcomes, characterized by a median overall survival (mOS) rarely exceeding 18 months and a 15% five-year survival (4, 5). In this context, immunotherapy has emerged as a promising therapeutic alternative, as the Checkmate 743 clinical trial described relevant improvements in terms of mOS in MPM patients after first-line treatment with nivolumab plus ipilimumab compared with platinum-based chemotherapy (18.1 months vs 14.1 months respectively) (6). This effect is related to the high prevalence of Programmed cell death ligand 1 (PD-L1) expression in MPM (28-56%) (7), which increases tumor susceptibility to this therapeutic blockade. However, PD-L1 importance in mesothelioma extends beyond this role and represents an independent predictor of unfavorable survival in MPM (8). Current prognostic assessment in patients with MPM is based on Cancer and Leukemia Group B (CALGB) prognostic score, which predicts poor clinical outcomes in individuals with poor performance status, non-epithelioid histology, male sex, low hemoglobin level, high platelet count, high white blood cell count, and high lactate dehydrogenase (LDH) levels (9). Nonetheless, the predictive factor of CALGB as a predictor of response to treatment is inconsistent in several studies (10). Therefore, this study aimed to explore the prognostic significance of PD-L1 in individuals with MPM to evaluate its complementation to CALGB score to enhance the therapeutic personalization of patients with this disease.

## 2 Methods

### 2.1 Patients

This retrospective study was conducted at the National Cancer Institute of Mexico from January 10, 2009, to December 31, 2019. This study was approved by the Institutional Ethics Committee (010/056/ICI) and Scientific Committees (CEI/656/10). Chemo-naïve patients with histologically confirmed malignant pleural mesothelioma (MPM) diagnosis were included. Tumor stage was determined according to the International Union Against Cancer tumor-node-metastasis 8th classification (11). Clinicopathological variables were retrieved from institutional database, including complete medical history, age, sex, performance status (PS), asbestos exposure, tumor stage, histologic subtype, treatment, and survival. To determine the prognostic value of PD-L1 expression, a cut-off point of 15% was determined by analyzing survival data using receiver operating characteristic curve (ROC) (area under the curve (AUC) = 0.70; 95% confidence interval (CI) 0.58-0.82, p = 0.003), as this cut-off value exhibited the highest sensibility (44.7%) and specificity (91.4%) for survival outcomes in our cohort (**Supplementary Figure 1**).

### 2.2 Tissue management

Tumor samples from 73 patients with MPM were processed and stored at room temperature. MPM diagnosis was confirmed by a pathologist specialized in oncological diseases. PD-L1 expression was determined using the VENTANA PD-L1 (SP263) Assay (Ventana Medical Systems, Tucson, AZ, USA) and detected using the OptiView DAB IHC Detection kit on a BenchMark IHC/HIS instrument. SP263 antibody was used as this is the standardized protocol for assessing PD-L1, and it counts with approval from the Food and Drug Administration (FDA) (12). PD-L1 tumor proportion score (TPS) was calculated as a percentage of at least 100 tumor cells with complete or partial membrane staining. PD-L1 positive samples were defined using a threshold of TPS ≥1%.

### 2.3 Therapeutic approach

Patients with advanced unresectable disease were included in this study. Therapeutic modalities included platinum-based chemotherapy, palliative radiotherapy (RT), combined chemoradiotherapy (CHT-RT), or best supportive care (BSC). Multimodality treatment (MMT) was defined as the combination of surgery (pleurectomy/decortication (P/D) or extra-pleural pneumonectomy), chemotherapy, and radiotherapy. Patients who underwent immunotherapy or other targeted agents were excluded from the study.

### 2.4 Statistical analysis

All statistical analyses were performed using SPSS (Statistical Package for Social Sciences) version 26.0 (SPSS Inc., Chicago, IL). For descriptive purposes, continuous variables were summarized as arithmetic means, and standard deviations (SD), categorical and ordinal parameters such as sex (male vs. female), clinical stage (III vs. IV), histological subtype (epithelioid vs. non-epithelioid), and dichotomized PD-L1 expression (<15% vs. ≥15%) were analyzed using the χ2 test or Fisher’s exact test. For survival curve analysis, all variables were dichotomized. Overall survival (OS) and progression-free survival (PFS) were analyzed using the Kaplan-Meier method, and comparisons among subgroups were analyzed using the log-rank test. Finally, we performed a multivariate analysis with a Cox proportional model to estimate the hazard ratios (HRs) with 95% CI adjusting for those variables, which were statistically significantly associated with survival in the univariate analysis. Statistical significance was set at p <0.05 based on a two-sided test.

## 3 Results

### 3.1 Study population and clinical characteristics

As shown in **Table 1**, this study included 73 malignant pleural mesothelioma patients with MPM, among which 68.5% were older than 60 years (n = 50) and 68.5% were male (n = 50). In addition, 43.8% (n = 32) had asbestos exposure and 53.4% (n = 39) had a smoking history. At diagnosis, 42.5% (n = 31) and 57.5% (n = 42) of the cases were classified as stages III and IV, respectively. Moreover, 78.1% (n = 57) of patients had an Eastern Cooperative Oncology Group (ECOG) Performance Status (PS) of 0-1. Epithelioid was the most common histological subtype in 84.9% of the cohort (n = 62).

**Table 1:** General characteristics of population according to PD-L1 expression.

Clinical characteristics	All % (n)	PD-L1 TPS <1% % (n)	PD-L1 TPS >1% % (n)	*P* value	PD-L1 TPS <15% % (n)	PD-L1 TPS >15% % (n)	*P* value
	100 (73)	57.5 (42)	42.4 (31)		71.2 (52)	28.7 (21)	
Sex							
Male	68.5 (50)	54 (27)	46 (23)		70 (35)	30 (15)	
Female	31.5 (23)	65.2 (15)	34.8 (8)	0.368^†^	73.9 (17)	26.1 (6)	0.732^†^
Age							
≤ 60 years	31.5 (23)	52.2 (12)	47.8 (11)		78.3 (18)	21.7 (5)	
> 60 years	68.5 (50)	60 (30)	40 (20)	0.530^†^	68 (34)	32 (16)	0.368^†^
Smoking history							
Absent	46.6(34)	50 (17)	50 (17)		58.8 (20)	41.2 (14)	
Present	53.4 (39)	64.1 (25)	35.9 (14)	0.224^†^	82.1 (32)	17.9 (7)	**0.029** ^†^
Wood smoke exposure							
Absent	71.2 (52)	59.6 (31)	40.4 (21)		75 (39)	25 (13)	
Present	28.8 (21)	52.4 (11)	47.6 (10)	0.571^†^	61.9 (13)	38.1 (8)	0.263^†^
Asbestos exposure							
Absent	56.2 (41)	51.2 (21)	48.8 (20)		70.7 (29)	29.3 (12)	
Present	43.8 (32)	65.6 (21)	34.4 (11)	0.217^†^	71.9 (23)	28.1 (9)	0.915^†^
ECOG PS							
0-1	78.1 (57)	59.6 (34)	40.4 (23)		75.4 (43)	24.6 (14)	
≥ 2	21.9 (16)	50 (8)	50 (8)	0.490^†^	56.3 (9)	43.8 (7)	0.134^†^
CALGB score							
1-2	19.2 (14)	71.4 (10)	28.6 (4)		92.9 (13)	7.1 (1)	
3-4	42.5 (31)	58.1 (18)	41.9 (13)		67.7 (21)	32.3 (10)	
5-6	38.4 (28)	50 (14)	50 (14)	0.415^‡^	64.3 (18)	35.7 (10)	0.133^‡^
Histological subtype							
Epithelioid	84.9 (62)	61.3 (38)	38.7 (24)		75.8 (47)	24.2 (15)	
Mix/Sarcomatoid	15.1 (11)	36.4 (4)	63.7 (7)	0.123^†^	45.5 (5)	54.5 (6)	**0.040** ^†^
Clinical stage							
III	42.5 (31)	61.3 (19)	38.7 (12)		67.7 (21)	32.3 (10)	

ECOG PS, Eastern Cooperative Oncology Group Performance Status. CALGB, Cancer and Leukemia Group B. PD-L1, Programmed Cell Death Ligand 1. TPS, tumor proportion score. PD-L1 cut-off value was determined by ROC curves, exhibiting the highest sensibility (44.7%) and specificity (91.4%) for survival outcomes in our cohort. Nominal variables were analyzed by ^†^Pearson Chi-Square test, except when small size of sample (n <5) required using ^‡^Fisher’s exact test. Significance was set at p < 0.05 (two-sided). Bold values mean statistically significant values (p<0.05).

### 3.2 PD-L1 expression

**Figure 1:**
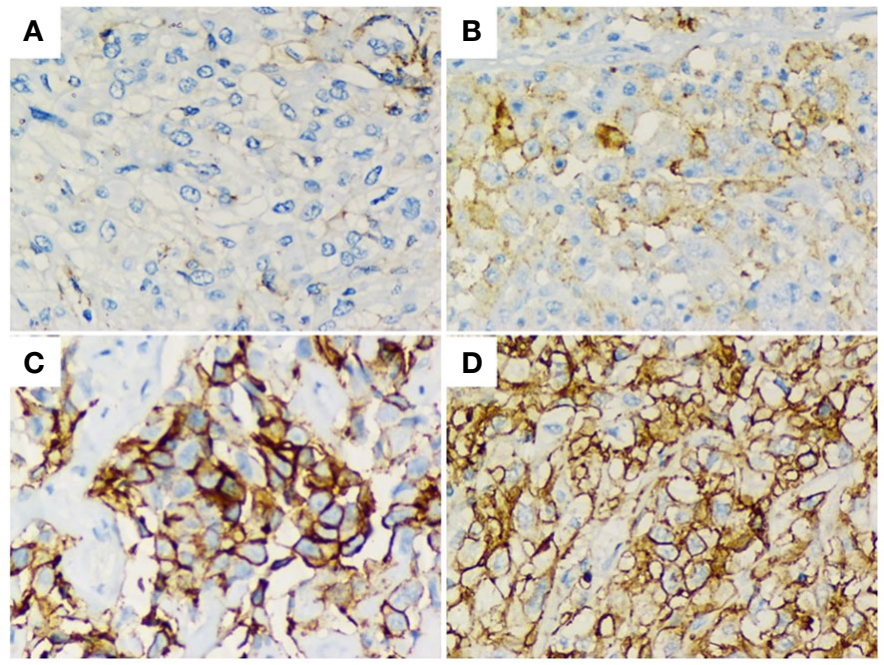
Immunohistochemical staining of PD-L1 (brown signal) from human MPM biopsies showing their median intensity; **(A)** 0/negative. **(B)** 1/weak. **(C)** 2/moderate. **(D)** 3/high. Magnification, x400. Staining index of PD-L1.

According to immunohistochemistry staining, PD-L1 intensity of expression was negative in 42.5% (31) and positive in 57.5% (42) of cases. Among positives, 31.5% (23) showed a low, 17.8% (13) intermediate, and 8.2% (6) high intensities (**Figure 1**). Regarding tumor proportion score, positive PD-L1 expression (TPS >1%) was present in 42.4% (n = 31) of individuals and absent (TPS <1%) in 57.6% (n = 42). In addition, they were categorized as high or low PD-L1 expressors based on a TPS cut-off value of 15%. Accordingly, 28.7% (n = 21) and 71.2% (n = 52) of patients harbored high and low PD-L1 TPS, respectively. Regarding the association of this biomarker with clinical characteristics, PD-L1^Low^ was associated with a smoking history (p=0.029) and epithelioid histology (p=0.04) (**Table 1**).

### 3.3 Progression-free survival

**Figure 2:**
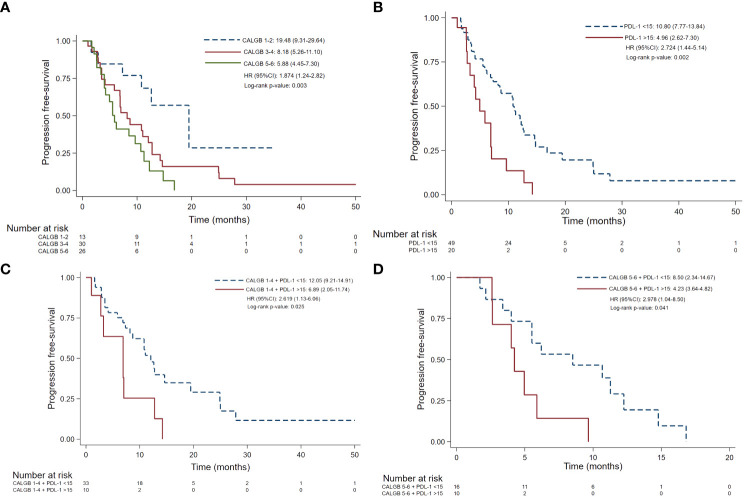
Progression-free survival according CALGB scales **(A)** PD-L1 TPS ≥15% **(B)** CALGB score 1-4 plus PD-L1 TPS >15% **(C)** CALGB 5-6 plus PD-L1 TPS >15% **(D)**. CALGB, Cancer and Leukemia Group **(B)** PD-L1, Programmed Cell Death Ligand 1. TPS, tumor proportion score. mPFS, median progression-free survival. Comparisons were performed using *log-rank test. Statistically significant p values were determined as p ≤ 0.05.

As displayed in **Table 2** and **Figure 2**, mPFS in the whole cohort was 8.70 (4.23 - 13.17) months. Clinical characteristics independently associated with PFS were CALGB 5-6 score (HR 1.94; 1.24-3.05; p=0.004) and PD-L1^Low^ (HR 0.43; 0.22 – 0.84; p = 0.014). Also, shorter mPFS was identified in individuals with CALGB 5-6 (5.88 vs. 8.18; HR 1.87; 95% CI 1.24-2.82, p=0.003), PD-L1 TPS >1% (4.9 vs. 10.8 months; HR 2.16; 95% CI 1.22-3.83, p=0.008) and PD-L1^High^ (4.96 vs. 10.8 months; HR 2.724; 95%CI 1.44-5.14, p=0.002).

**Table 2:** Progression-free survival according to clinicopathologic characteristics.

	mPFS (months)	95% CI	*P* value	Bivariate analysis HR (95% CI)	*P* value	Multivariate analysis HR (95% CI)	*P* value
**Overall:** 51	8.70	4.23 - 13.17					
Gender							
Male: 48	10.8	7.47-14.14		1.404 (0.78-2.50)	0.250		
Female: 21	7.03	5.85-8.20	0.246*				
Age							
≤ 60: 22	12.0	9.74-14.36		1.335 (0.71-2.48)	0.364		
> 60: 47	6.89	4.44-9.35	0.360*				
Smoking							
Absent: 31	6.20	3.57-8.83		0.610 (0.34-1.06)	0.083	0.596 (0.33-1.06)	0.080
Present: 38	12.0	10.33-13.78	0.079*				
WSE							
Absent: 49	10.6	7.15-14.19		0.964 (0.52-1.77)	0.907		
Present: 20	6.89	4.44-9.35	0.906*				
Asbestos exposure							
Absent: 40	8.50	4.89-12.16		0.769 (0.43-1.35)	0.365		
Present: 29	8.70	1.02-16.38	0.362*				
ECOG							
0-1: 55	7.32	5.43-9.22		0.457 (0.19-1.07)	0.073	0.450 (0.19-1.06)	0.069
≥ 2: 14	14.22	11.88-16.57	0.065*				
CALGB							
1-2: 13	19.48	9.31-29.64					
3-4: 30	8.18	5.26-11.10		1.874 (1.24-2.82)	**0.003**	1.949 (1.24-3.05)	**0.004**
5-6: 26	5.88	4.45-7.30	0.009*				
Histological subtype							
Epithelioid: 58	10.67	7.57-13.78		0.584 (0.28-1.21)	0.152		
Mix/Sarcomatoid: 11	4.23	2.76-5.71	0.146*				
Clinical stage							
III: 28	10.67	5.97-15.38		1.459 (0.82-2.59)	0.199		
IV: 41	8.18	4.52-11.83	0.194*				
PD-L1 TPS							
<1%: 39	10.8	9.01-12.60		2.168 (1.22-3.83)	**0.008**		
>1%: 30	4.96	1.94-7.98	0.006*				
PD-L1 TPS							
<15%: 49	10.80	7.77-13.84		2.724 (1.44-5.14)	**0.002**	0.439 (0.227-0.849)	**0.014**
>15%: 20	4.96	2.62-7.30	0.001*				

mPFS, median progression-free survival. CI, confidence interval. HR, hazard ratio. WSE, wood smoke exposure. ECOG PS, Eastern Cooperative Oncology Group Performance Status. CALGB, Cancer and Leukemia Group B. PD-L1, Programmed Cell Death Ligand 1. TPS, tumor proportion score. Comparisons were performed using *log-rank test. Statistically significant p values were determined as p ≤ 0.05. Bold values mean statistically significant values (p<0.05).

### 3.4 Overall survival

As shown in **Table 3**, mOS of all patients was 15.37 (12.38 – 18.36) months. Characteristics independently related to overall survival were asbestos exposure (HR 2.11; 95% CI 1.15-3.86, p=0.016) and PD-L1^Low^ (HR 0.13; 95% CI 0.06-0.28, p<0.001). Shorter mOS was identified in individuals harboring a PD-L1 TPS >1% (9.06 vs. 20.96 months; HR 2.72 95% CI 1.49-4.94, p=0.001), PD-L1^High^ (6.05 vs. 20.9 months; HR 6.87; 95% CI 3.4-13.89, p<0.001) and sarcomatoid/mix histology (7.72 vs. 16.95 months; HR 0.455 95% CI 0.21-0.95; p=0.036).

**Table 3:** Overall survival according to clinicopathologic characteristics.

	mOS (months)	95% CI	*P* value	Univariate analysis HR (95% CI)	*P* value	Bivariate analysis HR (95% CI)	*P* value
**Overall:** 47	15.37	12.38 – 18.36					
Sex							
Male: 50	15.37	10.83-19.92		1.102 (0.59-2.05)	0.759		
Female: 23	14.32	9.64-18.95	0.759*				
Age							
≤ 60 years: 23	16.95	7.46-26.44		1.307 (0.69-2.45)	0.405		
> 60 years: 50	14.48	8.17-20.80	0.403*				
Smoking history							
Absent: 34	13.14	7.59-18.68		0.567 (0.31-1.01)	0.056	0.769 (0.41-1.44)	0.412
Present: 39	16.95	14.44-19.45	0.053*				
WSE							
Absent: 52	14.48	10.04-18.93		1.021 (0.53-1.94)	0.949		
Present: 21	15.37	10.99-19.75	0.949*				
Asbestos exposure							
Absent: 41	15.37	11.15-19.54		1.763 (0.97-3.17)	0.059	2.110 (1.15-3.86)	**0.016**
Present: 32	14.32	6.91-21.73	0.055*				
ECOG PS							
0-1: 57	15.37	12.61-18.13		0.966 (0.47-1.96)	0.925		
≥ 2: 16	20.23	2.21-38.25	0.925*				
CALGB score							
1-2: 14	18.49	16.09-20.89		1.250 (0.83-1.88)	0.284		
3-4: 31	14.02	8.73-19.32					
5-6: 28	11.53	8.71-14.34	0.517*				
Histological subtype							
Epithelioid: 62	16.95	13.74-20.16		0.455 (0.21-0.95)	**0.036**	0.611 (0.27-1.36)	0.229
Mix/Sarcomatoid: 11	7.72	3.93-11.51	0.032*				
Clinical stage							
III: 31	16.95	7.45-26.44					
IV: 42	14.48	11.72-17.25	0.379*	1.301 (0.72-2.34)	0.381		
PD-L1 TPS							
<1%: 42	20.96	14.93-26.98					
>1%: 31	9.06	5.65-12.48	0.001*	2.720 (1.49-4.94)	**0.001**		
PD-L1 TPS							
<15%: 52	20.96	15.21-26.71		6.873 (3.40-13.89)	**<0.001**	0.139 (0.06-0.28)	**<0.001**
>15%: 21	6.50	4.30-8.70	<0.001*				

mOS, median overall survival. CI, confidence interval. HR, hazard ratio. WSE, wood smoke exposure. ECOG PS, Eastern Cooperative Oncology Group Performance Status. CALGB, Cancer and Leukemia Group B. PD-L1, Programmed Cell Death Ligand 1. TPS, tumor proportion score. Comparisons were performed using *log-rank test. Statistically significant p values were determined as p ≤ 0.05. Bold values mean statistically significant values (p<0.05).

### 3.5 Prognostic value according to CALGB plus PD-L1 expression

**Figure 3:**
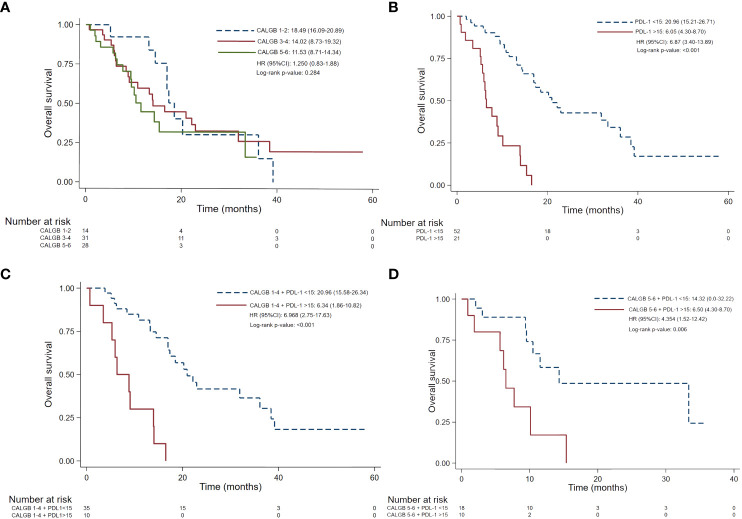
Overall survival according CALGB scales **(A)** PD-L1 TPS ≥15% **(B)** CALGB score 1-4 plus PD-L1 TPS >15% **(C)** CALGB 5-6 plus PD-L1 TPS >15% **(D)**. CALGB, Cancer and Leukemia Group **(B)** PD-L1, Programmed Cell Death Ligand 1. TPS, tumor proportion score. mOS, median overall survival. Comparisons were performed using *log-rank test. Statistically significant p values were determined as p ≤ 0.05.

As depicted in **Figure 3** and **Table 4**, CALGB 1-4 plus PD-L1^High^ demonstrates worse PFS (6.89 vs. 12.05 months, p=0.019) and OS (6.34 vs. 20.96 months, p<0.001) than CALGB 1-4 plus PD-L1^Low^. As well, CALGB 5-6 plus PD-L1^High^ demonstrated worse PFS (4.23 vs. 8.50 months, p=0.032) and OS (6.50 vs 14.32 months, p=0.003) than CALGB 5-6 plus PD-L1^Low^.

**Table 4:** Survival outcomes according to CALGB score and PD-L1 TPS.

CALGB score	mPFS (months)	95% CI	*p* value
1-2: 13	19.48	10.24-28.72	
3-4: 30	8.18	5.26-11.1	
5-6: 16	8.50	2.34-14.67	
5-6 + PD-L1 TPS ≥15%: 10	4.23	3.64-4.82	0.002*
CALGB score	mOS (months)		
1-2: 14	18.49	16.09-20.89	
3-4: 31	14.02	8.73-19.32	
5-6: 18	14.32	0.0-32.2	
5-6 + PD-L1 TPS ≥15%: 10	6.50	4.30-8.70	0.003*
CALGB plus PD-L1 TPS	mPFS (months)		
1-4 + PD-L1 TPS <15%: 33	12.05	9.21-14.90	
1-4 + PD-L1 TPS ≥15%: 10	6.89	2.05-11.74	0.019*
5-6 + PD-L1 TPS <15%: 16	8.50	2.34-14.67	
5-6 + PD-L1 TPS ≥15%: 10	4.23	3.64-4.82	0.032*
CALGB plus PD-L1 TPS	mOS (months)		
1-4 + PD-L1 TPS <15%: 35	20.96	15.58-26.34	
1-4 + PD-L1 TPS ≥15%: 10	6.34	1.86-10.82	<0.001*
5-6 + PD-L1 TPS <15%: 18	14.32	0.00-32.22	
5-6 + PD-L1 TPS ≥15%: 10	6.50	4.30-8.70	0.003*

CALGB, Cancer and Leukemia Group B. PD-L1, Programmed Cell Death Ligand 1. TPS, tumor proportion score. mPFS, median progression-free survival. mOS, median overall survival. Comparisons were performed using *log-rank test. Statistically significant p values were determined as p ≤ 0.05.

### 3.6 Prognostic value of CALGB plus PD-L1 expression according to histologic type

**Figure 4:**
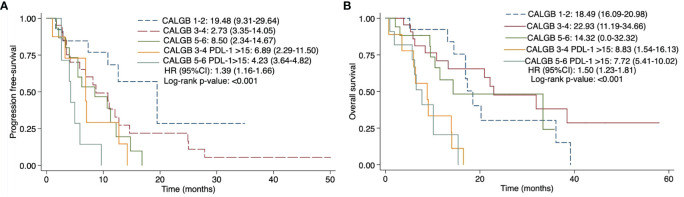
Progression-free survival **(A)** and overall survival **(B)** of CALGB 5-6 scale plus PD-L1 TPS ≥15% expression in epithelioid mesothelioma subtype. CALGB, Cancer and Leukemia Group **(B)** PD-L1, Programmed Cell Death Ligand 1. TPS, tumor proportion score. mOS, median overall survival. Comparisons were performed using *log-rank test. Statistically significant p values were determined as p ≤ 0.05.

As shown in **Figure 4**, a sub-analysis in epithelioid histology showed significant differences among individuals with different CALGB scores and PD-L1 expression (p=0.002). Subjects harboring a CALGB 5-6 score showed a worse mPFS (8.50 months; 95% CI 2.34-14.67) and mOS (19.51 months; 95% CI 13.44-25.58) than those having a CALGB 3-4 (mPFS 8.70 months; 95% CI 3.35-14.05 and mOS 26.18 months; 95% CI 18.66-33.70) or CALGB 1-2 scores (mPFS 19.48 months; 95% CI 9.31-29.64 and mOS 17.90 months; 95% CI 16.23-19.57). As well, CALGB 5-6 score plus PD-L1 TPS>15% predicted a worse mPFS (6.65 months; 95% CI 3.64-4.82) and mOS (15.37 months; 95% CI NR-NR) than those harboring CALGB 3-4 score plus PD-L1 TPS >15%, which displayed better mPFS (6.89 months; 95% CI 2.29-11.50) and mOS (14.02 months; 13.87-14.18).

## 4 Discussion

This study places PD-L1 as a promising parameter for complementing CALGB score, ultimately improving its predictive assessment of progressive disease and death in patients with MPM during precision oncology era. PD-L1 prevalence in our cohort (42.4%) is higher than that reported in European cohorts (13), likely derived from divergent exposures to risk factors; for instance, frequencies of smoking history and asbestos inhalation are similar among this cohort and previous Latin American reports (14). As well, PD-L1 expression harbor similar clinical characteristics in other cohorts than in ours, commonly associated with sarcomatous subtype, poorly differentiated histology, and poor prognosis (12, 13).

Regarding PD-L1 staining intensity, other populations have reported higher prevalence of negativity to PD-L1 expression (75-79%), but in a similar way to our study, PD-L1-positive individuals showed a higher proportion of patients with weak staining [(13, 15). Moreover, prognostic assessment based on PD-L1 expression is limited by a lack of consensus regarding its most optimal expression cut-off value affecting clinical outcomes; particularly, our results show a higher prevalence of PD-L1 TPS >1% than other populations (13, 15, 16), which also demonstrated consistent prognostic significance in terms of PFS (10.8 vs 4.96 months; p=0.006) and OS (20.96 vs 9.06 months; p=0.001) compared to other studies; for example, Desage et al. (13) correlated PD-L1 TPS >1% expression with shorter mOS in 77 MPM patients undergoing chemotherapy (4.79 vs 16.3 months). Similarly, a meta-analysis of 16 retrospective studies identified a worse OS in 1899 MPM individuals with PD-L1 expression over 1-5% cut-off value (17). Furthermore, similar cut-off values have been proposed in literature for determining high PD-L1 expression to those set in this study; for example, Brcic et al. (15), identified in a multicenter study worse OS outcome in 203 individuals with MPM (6.05 vs 20.9 months) exhibiting high PD-L1 expression (TPS >10%). Therefore, PD-L1 represents a poor prognostic factor in multiple studies.

These findings suggest a relevant biological transcendence of PD-L1, which focuses on its intrinsic immunosuppressive role, impairing cytotoxic cell responses and promoting tumor progression (18). This study did not evaluate tumoral immune infiltration, constituting an important limitation in this regard, but extensive research has revealed that PD-L1 may not be a feasible biomarker for predicting tumoral infiltration; Cedres and cols (13). did not find a significant relationship between Tumoral Infiltrating Lymphocytes (TILs) and PD-L1 expression in MPM patients, but those with positive (TPS >1%) PD-L1 expression showed higher presence of CD8+ or CD4+ lymphocytes (13). Complementary, other immune factors may also be involved in MPM progression are enriched infiltration of M2 macrophages, along with impaired T CD8+ CD163+ cell functionality, caused by macrophage-released arginase, IL-10, and TGF-β, and tumor-associated fibroblasts (10), which are known to create an immunosuppressive microenvironment (19). Other biomarkers are currently studied for their prognostic significance in mesothelioma, such as GLUT-1, COX-2, p27 (9),, CDKN2Adeletion (20), neutrophil-to-lymphocyte ratio (NLR), platelet-to-lymphocyte ratio (PLR), monocyte-to-lymphocyte ratio (MLR) (19), and low RRM1 and ERCC1 (21), but these have not been explored in large-sampled studies to completely generalize their usage in clinical practice. Furthermore, novel prognostic scales have been created in recent years considering the clinical and molecular aspects of MPM, showing worse survival outcomes in patients with certain oncogenic alterations (22), cellular adherence markers (23), and clinical stages (24).

Although PD-L1 has been widely demonstrated to affect survival outcomes in mesothelioma, it is not entirely predictive of response. PD-L1 (TPS>15%) predicts a poorer prognosis in addition to CALGB (any grade) than using CALGB alone; thus, PD-L1 may improve predictive accuracy of CALGB in MPM patients (8, 21). Addition of PD-L1 expression to CALGB score also demonstrated to predict poorer survival outcomes among individuals with epithelial histology, even after previous studies have not demonstrated significant differences in PD-L1 expression between epithelial and non-epithelial histologies of MPM; thus, these findings suggest that complementing CALGB score with PD-L1 expression may harbor an unveiled biological relationship among them which demands further study. Moreover, although CALGB is part of the current prognostic assessment of malignant pleural mesothelioma, its design adjusts to classical therapeutic approaches and results inaccurate for emergent immunotherapy-based regimens, which demands the inclusion of immunological biomarkers, such as PD-L1. Furthermore, survival outcomes predicted for different CALGB groups in this study were slightly different from those previously reported in different cohorts, which may be attributed to variations in their therapeutic management (25). In this regard, our cohort underwent standard treatment with cisplatin and pemetrexed, but better clinical outcomes have been described in previous evidence by combining chemotherapy with bevacizumab (19), pembrolizumab (26), gemcitabine as continuous infusion (27), liposomal doxorubicin (28), or substituting it with ipilimumab plus nivolumab (6). In contrast, lack of benefit has been identified in unresectable epithelioid MPM after monotherapy with CTLA-4 (29) as second-line of treatment or VEGF (30) blockers as first-line approach. CALGB has demonstrated inconsistent results for estimating prognosis during follow-up of MPM individuals undergoing second-lines of treatment; for instance, Dudek and cols (31). identified non-significant differences between MPM subjects previously treated with pemetrexed-carboplatin, who then underwent maintenance with pemetrexed.

This report has some limitations, among which the most representative are its retrospective nature and small sample size, which may affect generalizing its findings to populations with different characteristics. As well, derived from the immunosuppressive role of PD-L1 in tumoral biology, the lack of measurement of infiltrating immunologic cells in tumoral microenvironment represents an important limitation in the comprehension of the underlying mechanism behind our results. Then, these findings support the future performance of larger-sampled prospective studies exploring the immunologic microenvironmental implications of complementing CALGB with PD-L1 expression.

## 5 Conclusion

PD-L1 addition to CALGB scores represents an independent prognostic factor for shorter PFS and OS in advanced-stage MPM patients, thereby expanding the predictive accuracy of CALGB alone. The main contribution of this study is highlighting the need to design novel prognostic scales, including immune biomarkers, to promote better therapeutic personalization in Latin American individuals with this disease.

## Data availability statement

The raw data supporting the conclusions of this article will be made available by the authors, without undue reservation.

## Ethics statement

The studies involving humans were approved by Institutional Ethics Committee and Scientific Committees. The studies were conducted in accordance with the local legislation and institutional requirements. Written informed consent for participation was not required from the participants or the participants’ legal guardians/next of kin because Informed consent was waived because this was a retrospective study.

## Author contributions

OA: Conceptualization, Writing – original draft, Writing – review & editing. AA-S: Formal Analysis, Methodology, Writing – review & editing. LC-M: Methodology, Writing – original draft, Writing – review & editing. NH-P: Formal Analysis, Methodology, Writing – original draft, Writing – review & editing. DV-L: Methodology, Writing – review & editing. WM-M: Conceptualization, Writing – original draft. DM-K: Writing – original draft, Writing – review & editing. LC-R: Supervision, Writing – original draft, Writing – review & editing. CM: Conceptualization, Supervision, Writing – original draft, Writing – review & editing. AC: Writing – original draft, Writing – review & editing. CP-P: Project administration, Writing – review & editing. SS: Conceptualization, Writing – original draft, Writing – review & editing.
