# Supplementary material for: PD-L1 expression complements CALGB prognostic scoring system in malignant pleural mesothelioma
Source: Front Oncol. 2023 Dec 4;13:1269029. doi: 10.3389/fonc.2023.1269029 (PMC10725960; doi:10.3389/fonc.2023.1269029)
Supplement: Supplementary file 1 [file DataSheet_1.pdf]

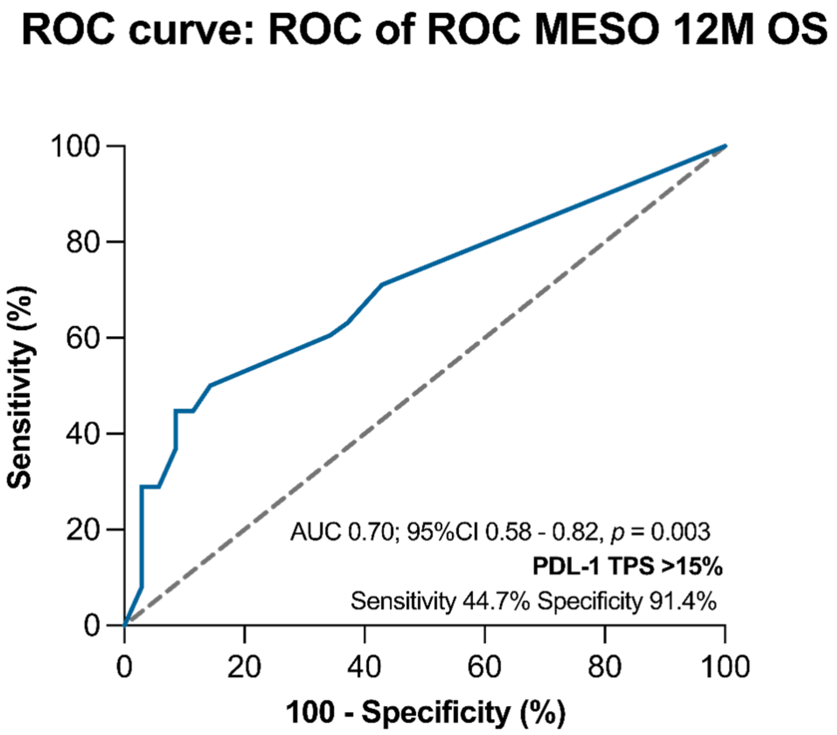

Supplementary figure 1. ROC curve for determining high PD-L1 TPS percentage. PD-L1, Programmed Cell Death Ligand 1. TPS, Tumor Proportion Score. AUC, Area under curve. Statistical significance was set at $p < 0.05$.
